# Possible Evidence for a Fall in the Prevalence of High-Functioning Pervasive Developmental Disorder with Age?

**DOI:** 10.1155/2011/325495

**Published:** 2011-06-19

**Authors:** M. Balfe, D. Tantam, M. Campbell

**Affiliations:** ^1^University of Huddersfield, Huddersfield HD1 3DH, UK; ^2^School of Health and Related Research, The University of Sheffield, Sheffield SL 4DA, UK; ^3^Developmental Psychiatry Section, University of Cambridge, CB2 8AH, UK; ^4^School of Health and Related Research, The University of Sheffield, Scheffield SL 4DA, UK

## Abstract

A survey was undertaken to investigate the prevalence of high-functioning pervasive developmental disorder (HFPDD) in a community sample of teenagers and adults aged 13 and above in the city of Sheffield, UK. 112 possible and definite cases were found, of whom 65 (57%) had a previous diagnosis. The detected prevalence of possible or definite HFPDD was found to be 0.24 per 1000 of the population of Sheffield city aged 13 or over, but the prevalence by year of age fell from a maximum of 1.1 per 1000 in the group aged 13 to 14 years old (1 young adult in every 900 in this age group) to 0.03 per 1000 in the over 60s (1 person in every 38500 in this age group). The results of this study are preliminary and need follow-up investigation in larger studies. We suggest several explanations for the findings, including reduced willingness to participate in a study as people get older, increased ascertainment in younger people, and increased mortality. Another contributory factor might be that the prevalence of high-functioning pervasive development disorder may decline with age. This raises the possibility that AS symptoms might become subclinical in adulthood in a proportion of people with HFPDD.

## 1. Introduction

The UK Government has recently (March 2010) published a strategy for adults with autism (Fulfilling and rewarding lives) building on the Autism Act of 2009, and the National Audit Office report *Supporting people with autism through adulthood *[1]. The “second strand of the strategy” is to develop a “clear, consistent pathway for [the] diagnosis of autism.” The strategy also calls for estimates of the numbers of adults with autism.

Currently the diagnosis and management of adults with an autism spectrum disorder (an ASD) in combination with an intellectual disability is the responsibility of learning disability teams, who have considerable, and well-established, experience. Responsibility for the diagnosis of people with an ASD who do not have an intellectual disability is much less clear, although a recent report of the Royal College of Psychiatrists in the UK indicated that it can best be carried out by community mental health teams, not least because many adults with an ASD seeking diagnosis may have a comorbid psychiatric disorder. Trainee psychiatrists and clinical psychologists have been expected to be familiar with the criteria for the diagnosis of autism for years (unpublished correspondence to the second author from the board of examiners of the UK Royal College of Psychiatrists, and the British Psychological Society), but people seeking a diagnosis of ASD are regularly turned away from mental health teams because they lack training.

Community mental health teams may also argue that they are not commissioned to provide diagnosis or management, but this is likely to change once the strategy is implemented. Community mental health teams are hard pressed in the UK and everywhere in the developed world. Team members may be reluctant to accept responsibility for adults with an ASD but no learning difficulty considering the rising prevalence of children identified with an ASD over the last decade, with prevalence estimates currently reaching those of schizophrenia, that is, 1% of the population (Baird et al.). This rise in reported prevalence has been much greater in those without an intellectual disability, who now account for about half of those with a diagnosis of an ASD. 

It is commonly assumed that these prevalence figures, which have all been based on surveys of children, also apply to adults. If that were so, then 0.3% of adults with no intellectual disability would have an ASD and any of them who have not previously had a diagnosis might turn to the community mental health team for a diagnostic assessment. Inevitably some people who were diagnosed as children may want the diagnosis to be reviewed and may also turn to the community mental health team for services or advice. 

How big a demand will this be? Are colleagues right to fear that they will be inundated, as some do?

We have surveyed all residents of Sheffield aged 13 and over who may have or are known to have an ASD but do not have an intellectual disability, and we consider here estimates of the demand for diagnosis, for treatment of comorbid psychiatric disorder, and for social care based on our findings.

## 2. Background

### 2.1. Diagnosis

High-functioning pervasive developmental disorder (HFPDD), also known as Asperger's syndrome, is an autistic spectrum condition characterised by “qualitative abnormalities of reciprocal social interaction that typify autism, together with a restricted, stereotyped, repetitive repertoire of interests and activities” [2]. This definition, taken from the International Classification of Disease, is accompanied by a description of the disorder, which includes that suggestion that “at least some cases represent mild varieties of autism” but the definition of HFPDD in the most recent Diagnostic and Statistical Manual of mental disorders [3] states that a diagnosis of HFPDD cannot be made if a diagnosis of autistic disorder is applicable. It has been pointed out that the application of this criterion makes it almost impossible to receive a diagnosis of HFPDD using DSM-IV criteria [4]. No criteria have been found to separate autistic disorder and HFPDD [5–9] other than symptom severity. When differences are found they can often be attributed to the fact that children with language impairment are included in the autistic disorder group and excluded from the HFPDD group [10], but language impairment often remits in late childhood in the more able group. The term “high-functioning autistic disorder” is also used in the USA for those people with autistic disorder who have less severe symptoms and we have assumed in this study that HFPDD, Asperger's syndrome, Asperger's disorder, and high-functioning autistic disorder are synonymous and that they are differentiated from other autistic disorders by an absence of current language or cognitive impairment. We have therefore used HFPDD and high-functioning autism (HFA) interchangeably and have included in our study all those with a pervasive developmental disorder in the absence of an intellectual disability.

### 2.2. Prevalence

For decades autism (of all subtypes) was considered to be a rare condition with a low prevalence of approximately 2–4 per 10,000 children [11]. Recent epidemiological research suggests that the prevalence of autistic spectrum disorders may be much higher than previously thought [12], possibly up to 20–30 per 10,000 [13]. It is unclear whether autism spectrum disorders are themselves increasing in prevalence; however, it is thought that changes in diagnosis and criteria [14], together with improved awareness of ASDs among diagnostic teams and clinicians, have contributed to this increase [13].

Within the context of this overall increase, the prevalence of HFPDD is considered to be lower than that of other autistic disorder subtypes [13], possibly as low as 2.5 per 10,000 if conservative estimates are employed [15]; though, some studies have found a higher prevalence of HFPDD of up to 9.2 per 10,000 [14]. Males predominate individuals diagnosed with HFPDD more than they do individuals diagnosed with traditional autism [14]. There are alternate explanations for the lower prevalence rate of HFPDD: one may be that HFPDD is simply less common than other forms of ASD; it may also be that it is underrecognized in children with normal intelligence, whose intellectual abilities can disguise their underlying PDD [16]. It may also be that the research base is simply lacking due to the fact that HFPDD was only recently included as a diagnostic category in DSM and ICD-10 [17]. 

Most ASD prevalence studies base their estimates on figures derived from child populations [12, 15]. This makes practical sense; childhood is when ASD behaviors are likely to manifest themselves most clearly; children are also subject to surveillance by a range of health, education, and social services that can pick up signs of ASD. The consequence of this child-centered approach to prevalence, however, is that there is very little research into estimating prevalence in adults with HFPDD. This is potentially an important absence; even within child populations estimates of HFPDD can vary depending on when children are assessed—estimates obtained in younger samples might underestimate the prevalence of HFPDD compared to estimates derived from children in their early teens [18]. It may well be that HFPDD prevalence can also change as children grow older, either increasing or, perhaps, decreasing. However, since few studies to date have examined adult HFPDD prevalence, it is unknown whether childhood prevalence figures stay static into adulthood or changes.

## 3. Methods

### 3.1. Sample

We surveyed adolescents aged 13 and above along with adults living in the city of Sheffield who had either received a clinical diagnosis of a high-functioning autism spectrum disorder/pervasive developmental disorder or who met research criteria for high-functioning ASD, that is a characteristic developmental profile plus current social and communicative impairment using a standard observational scale.

To recruit adolescents we wrote to all headmasters in Sheffield asking them to draw the study to the attention of school educational needs coordinators and the pupils for whom they were responsible. Because of ethical considerations we could not use school records or obtain information from the local education authority. We sent letters to every general practice in Sheffield, to all neurologists, paediatricians and community paediatricians, and psychiatrists, inviting them to bring the study to the attention of any of their patients who had, or whom they suspected of having, high-functioning pervasive developmental disorder (also known as Asperger, syndrome or high-functioning Autism).

To increase the recruitment of adults and older adolescents, especially those without a previous diagnosis, we designed posters to invite people to participate if they had life-long difficulties in making friends. We placed posters in supermarkets, shops, cinemas, post offices, libraries, pubs, workplaces, student noticeboards, employment assistance agencies, and GP practices throughout the city. We wrote articles for the local Sheffield Star and Sheffield Telegraph (the local newspapers with the highest circulation in Sheffield), and gave an interview on Radio Sheffield about the study; this interview was subsequently hosted on the main BBC news website. We recruited respondents through the Sheffield Asperger's Parents Action Group (SAPAG). We placed information about the project to the Sheffield “Prospects” office (the employment agency for people with autism in Sheffield) and sent information about the study to social workers, care workers, and disability workers throughout Sheffield.

### 3.2. Measures

All potential participants were asked about place of residence and age. Children younger than 13 years, those living outside the city of Sheffield, and those who had previous psychometric testing showing that they had an IQ below 70 were excluded. The remainder were sent the Autism Spectrum Quotient (AQ), a 50-item autism screening instrument developed by Baron-Cohen et al. [19]. Baron-Cohen et al. suggest that 26 is a valid cut-off point for distinguishing likely autistic behaviour from non-autistic [20]. Participants were also asked for additional consent to be interviewed and for their parents to be interviewed. They were assessed using a specially devised observational scale of communicative impairment [21] in place of the more usual ADOS that had not been validated in adults [22] at the time of the survey, and the Wechsler Abbreviated Scale of Intelligence (WASI) [23]. Their parents were interviewed using the revised Autism Diagnostic Interview (ADI-R) [24] by a research worker trained in its use who was unaware of the participant's AQ scores at the time of the interview. 

The North Sheffield Local Research Ethics Committee granted the project ethical approval and all respondents gave written informed consent.

## 4. Results

151 people from Sheffield contacted the research team and were sent an AQ and a questionnaire. The questionnaire asked respondents for their address in Sheffield and whether or not they had a clinical diagnosis of AS. 120 (out of 151) AQs were returned. Six of these participants were excluded from the prevalence study because their AQ scores were below threshold and they had no previous diagnosis of PDD, and 2 were excluded because their addresses were outside the city boundary.

Only 23 participants (out of the 120 who returned AQs) agreed to be interviewed and to complete a WASI, and only 36 allowed their parents to be interviewed although, as no parent refused, ADI-R data were obtained on all of these 36 participants. Twenty of the completed WASIs were scored above 70: the three who scored below 70 had been assessed previously by a clinician as diagnosed as having high-functioning autism.

Of the 36 participants whose parents completed the ADI-R, 26 had previously been given a clinical diagnosis of HFPDD disorder and there was good concordance between a diagnosis and having an ADI-R score above threshold. We therefore felt confident about relying on clinical diagnosis as an indication of “definite HFPDD” (see Table 1). 

Although there was 72% agreement between an above threshold ADI-R score and an AQ above 26 (Table 2), the kappa was zero as there was a substantial degree of false positives and false negative findings using the AQ. 

Because of the good concordance between a clinical diagnosis of HFPDD (usually made by a local, specialist ASD team) and ADI-R score above threshold, we included all respondents who reported that they had a clinical diagnosis. There were 65 participants in this definite group. In addition there were 49 participants with AQ scores over threshold (score = 26 or above) that we considered to “possibly” have ASD although we recognize that this criterion probably over-estimates the numbers with ASD. 

We calculated the prevalence using the rates of Sheffield residents in each age and gender group available from the national census (Office of National Statistics, accessed 2007) in the definite group (Tables 1 and 2).

**Figure 1:**
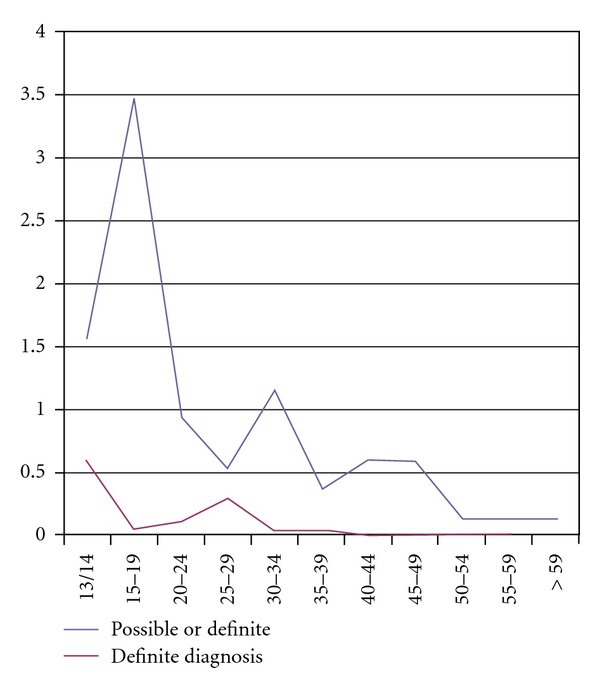
Age-related prevalence of HFPDD in males per 1000 population of the city of Sheffield in that age group.

We calculated prevalence for each age group. There was a fall with age for participants, for women (see Table 3) but particularly for men (see Table 4 and Figure 1).

We also plotted the frequency of participants with a definite or probable diagnosis of HFPDD by postal district of residence. This showed that participants from many districts of Sheffield were underrepresented and that the largest number of participants lived in Sheffield 8, followed by S11 and S6. These areas are described in the Acorn classification of UK postcodes as including the wealthy or the comfortably off.

## 5. Discussion

### 5.1. Summary

The prevalence of HFPDD has varied from study to study, with more recent studies tending to report greater prevalence averaging out at about 0.6% for the prevalence of all autistic spectrum disorders, including AS [15]. The rate of Asperger's disorder in one community study of 3–10-year olds, where the prevalence of ASD was found to be 0.67%, was 0.27% [15]. Many adults will have missed diagnosis during their childhood [20]. Most of the studies that have attempted to calculate prevalence have done so using children (Fombonne and Tidmarsh, 2003). We conducted a study to examine the prevalence of HFPDD in an adult and adolescent (aged 13 and above) community sample in Sheffield in the UK. Sheffield is the fourth biggest city in England, with a population of 435,000 13 and older (figures taken UK Office of National Statistics 2001 population figures for Sheffield). We considered the lifetime risk for all those 13 and above.

### 5.2. Strengths and Weaknesses

This is the first community-based epidemiological study to attempt to map the prevalence of HFPDD in adults. We did not rely on previous diagnosis or professional ascertainment but invited members of the public to come forward through a public awareness campaign. We were therefore able to capture Sheffield residents who had never been diagnosed, and in the event, almost half of the adolescents and adults that were included were not previously known to have an autistic spectrum disorder. 

Including people not previously known to services as having an ASD was a strength of our recruitment method, but it also the weakness that some people with AS may not have heard about the study, or if they did, may not have been willing to come forward. It is likely that there was a selection bias influencing those participants who did come forward and, therefore, that we have underestimated the prevalence of AS. For instance there is also an underrepresentation of nonwhite groups in our study, and an uneven geographical distribution of the addresses of our respondents with many more respondents from south rather than north Sheffield. 

Unlike several previous epidemiological prevalence studies that were conducted with children [16], we were not able to intensively screen all study respondents.

### 5.3. A Possible Fall in Prevalence with Age?

The ICD-10 description of Asperger's syndrome includes the following: “There is a strong tendency for the abnormalities to persist into adolescence and adult life…” [2, page 258]. DSM-IV is more definite, stating firmly that “Asperger's disorder is a continuous and lifelong disorder” [3, page 82]. The implications of this are far-reaching. For one, a child diagnosed with HFPDD can expect, as can their family, that there will be no escape from social impairment, ever. Asperger was not of this opinion himself, arguing that autistic psychopathy had a “good prognosis.” It is difficult to know on what evidence the assumption that it is a lifelong disorder is based. In the Goteborg study mentioned earlier [14] the prevalence rate in the 1989–1994 age cohort (children who were between 7 and 12 at the time of the sample) was 6.8 per 1000, the rate in children from 13 to 18 was 15.1 per 10000 but the rate in the adults (aged 19 to 24) was 7.0 per 10000. A similar pattern of a falling prevalence in age, although of all children with an ASD and at an earlier age, was reported in a much larger study in the the USA [25].

The overall prevalence rate that we found, 2.4 per 10,000, is below the prevalence rate of 9.5 per 10 000 suggested by some recent estimates of the rate in children [16] and below that of the adult rate of 7 per 10 000 reported by Gillberg et al. [14]. However, like Gillberg et al., we found that rates fell with chronological age and the mean prevalence in our youngest two age groups, of 13- and 14-year olds, was 11 per 10 000 and therefore comparable to other studies. 

The fall in prevalence with age was also shown by the group who had previously received a clinical diagnosis, suggesting that it might not be an artefact of self- or parent overrecognition of HFPDD. It was also found in the not diagnosed group, too, suggesting that it was not a consequence of the diagnosis being available in young people consulting child and adolescent mental health services or in an increase in rates of diagnosis made by these services over the last ten years (see Figure 1).

### 5.4. Alternative Explanations for Results

The numbers in the cells are small, and there are other possible explanations of this apparent fall. One is that parents are instrumental in discovering about, and facilitating their children's participation in, community activities, including surveys of this kind. Adults may therefore be less likely to participate because they are less influenced by, or less in contact with, their parents, although our survey also showed that many of our participants continued to live with their parents [26]. Other possible reasons for attrition include mortality and institutionalization and therefore inaccessibility to a community-based survey. Some individuals with HFPDD may simply have not been aware that the project was taking place, despite our best efforts; mapping respondents postcodes reveals that most respondents came from a few central areas of Sheffield and that outlying postal districts were underrepresented. 

Adults may also be reluctant to take part in a study such as this for fear of being labelled as having a disability or a mental health problem. We had some evidence of the latter in that some of the women who participated in the early phases of the study were unwilling to be interviewed because they had met new partners whom they did not want to tell of their (possible) condition. 

The likelihood that children with HFPDD are diagnosed has increased substantially since HFPDD was first officially recognized. This would be reflected in an apparent reduction in the prevalence of older people with known HFPDD in the general population and is perhaps the most economical explanation of the apparent fall in prevalence, where it not for the fact that the fall was also seen in the undiagnosed group, who would not be influenced by this ascertainment effect.

**Figure 2:**
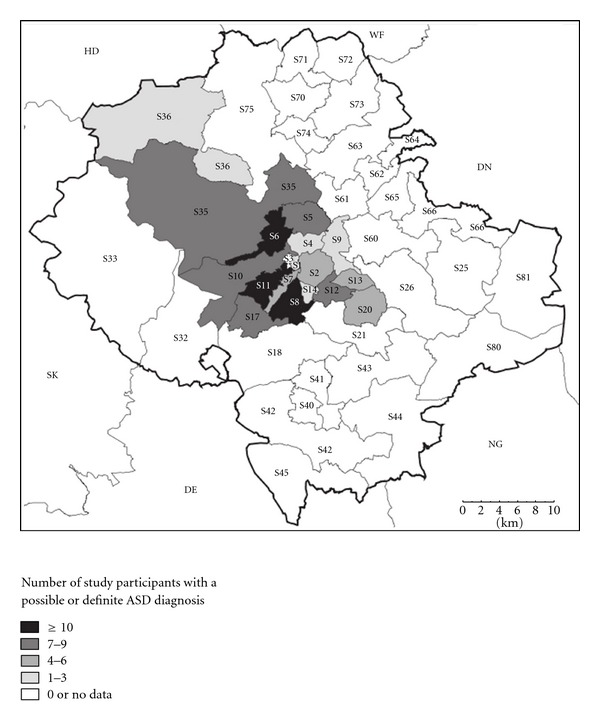
Spatial distribution by postcode of HFPDD cases in the city of Sheffield (greater Sheffield postcodes outside city are shown as white).

It is likely that this study has not captured everyone with high-functioning autism in Sheffield (previous community prevalence studies of children acknowledge that it is highly unlikely that everyone with AS in a community will be captured by one study [27]. The spatial distribution of diagnosis suggests that ascertainment was highest in the wealthier parts of the city (see Figure 2). We do not propose it as sufficient evidence that HFPDD/ HFA may remit in adult life, although we do consider that the evidence provides a hypothesis that it may do so.

### 5.5. Implications

Despite the limitations of this study, ours is the first community study of HFPDD in adults and it suggests that there may be a fall in prevalence of HFPDD with age, consistent with Asperger's original statement that “autistic psychopathy,” as he termed HFPDD disorder, has a “good prognosis” [28]. This finding is perhaps supported by the fact that no person older than 65 decided to participate in this study (although one of us—DT—has seen several people in their 70s in his clinic suggesting that people of this age may still seek a diagnosis). The possibility that HFPDD remits with age should not be a surprise since it is exactly the pattern seen in attention deficit hyperactivity disorder with which HFPDD has many overlaps [29].

Our findings need replication in larger longitudinal studies but if, true, have important clinical implications. One is that efforts to increase the rate of this spontaneous remission are worthwhile. People with HFPDD are often satisfied with the help that they receive from health services [26]. Unfortunately, in many cases very little help is available for people with high-functioning pervasive developmental disorder [30]. 

Another implication is that we may need to consider more carefully whether HFPDD is a more variable disorder than is normally thought. Although its basis is neurodevelopmental, it is possible that, like other neurodevelopmental conditions, the functional impairment varies according to social, emotional, or cognitive demand. Childhood is a time of considerable social and emotional demand, and it is possible that the remission of AS in adulthood is partly a consequence of the remission of this social demand. The clinical significance of this would be that, if true, then it could be expected that AS might apparently “recur” at some later stage when there is a surge of further demand.

We do think that the results of this study are evidence that the fear that adult mental health services will be overwhelmed by a tide of demand if they accept responsibility for the recognition and treatment of HFPDD is groundless. Most of the participants in the present study had already received a diagnosis. So even if screening were instituted, based on the findings of this study the demand for diagnosis would be increased by less than a factor of 2. Followup of our participants often indicated that many did not want further services and were satisfied with the services that they had received. Other participants did not want to receive a formal diagnosis. So it is likely that the true immediate demand on services would be less even than this.

Finally, we would acknowledge again that, while our figures may not be completely representative of the universe of people with HFPDD, they may more accurately reflect those for whom AS causes significant social impairment. Our figures do make a strong case for having more definitive surveys, both to see if our findings are replicated and because if AS does become less prevalent with age there are important and obvious implications for services and for the science of HFPDD aetiology. It would be useful if future studies employed a door-to-door method of ascertainment (although even this will miss people with HFPDD who are homeless, in institutions, or living alone and reluctant to answer the door) to assess prevalence (since there is no equivalent in adult life to the school record of pupils with difficulties), but also to have more up-to-date methods of assessing social impairment which reflected not just objective role performance (e.g. working, living independently, and so on) but also subjective assessment of the quality of social inclusion and participation.

## Figures and Tables

**Table 1:** The concordance of clinical diagnosis and ADI-R scores above threshold.

	Previous diagnosis of HFPDD
ADIR+	24
ADIR−	2

**Table 2:** The correlation between ADI-R and AQ.

	AQ ≥ 26	AQ < 26
ADIR+	25	5
ADIR−	5	1

**Table 3:** Distribution of females with possible or definite diagnosis of HFPDD by age.

	AQ is between 26-31 (inclusive) and no clinical diagnosis	AQ ≤ 32 and previous clinical diagnosis	AQ ≥ 32 and previous clinical diagnosis	AQ ≥ 32 and no previous clinical diagnosis	Total included with possible or definite ASD
13 to 14	1			1	2
15–19	1		1		2
20–24					
25–29	1	2	2		5
30–34	1	3	1	3	8
35–39				3	3
40–44				2	2
45–49				1	1
50–54	1				1
55–59				1	1
60 and over	1			1	2

**Table 4:** Distribution of males with possible or definite diagnosis of HFPDD by age.

Age group	AQ =26–31and no clinical diagnosis	AQ ≤ 32 and previous clinical diagnosis	AQ ≥ 32 and previous clinical diagnosis	AQ ≥ 32 and no previous clinical diagnosis	Total included with likely or confirmed ASD
13 to 14	2	5	4	1	12
15–19	1	10	11	4	28
20–24	3	1	2	2	8
25–29		3	2	2	7
30–34		3	8	3	14
35–39	1	1	1	1	4
40–44	3		2	1	6
45–49			2	3	5
50–54	1				1
55–59	1				1
60 and over			1		1
